# In Situ Synergistic Catalysis Hydrothermal Liquefaction of Spirulina by CuO–CeO_2_ and Ni–Co to Improve Bio-oil Production

**DOI:** 10.1021/acsomega.2c05619

**Published:** 2023-02-20

**Authors:** Yanghao Meng, Hongbiao Du, Shuai Lu, Yanwei Liu, Jinglai Zhang, Hualong Li

**Affiliations:** † School of Environment & Natural Resources, Renmin University of China, Beijing 100872, China; ‡ Institute of Intelligent Machines, Hefei Institutes of Physical Science, Chinese Academy of Sciences, Hefei, Anhui 230031, China

## Abstract

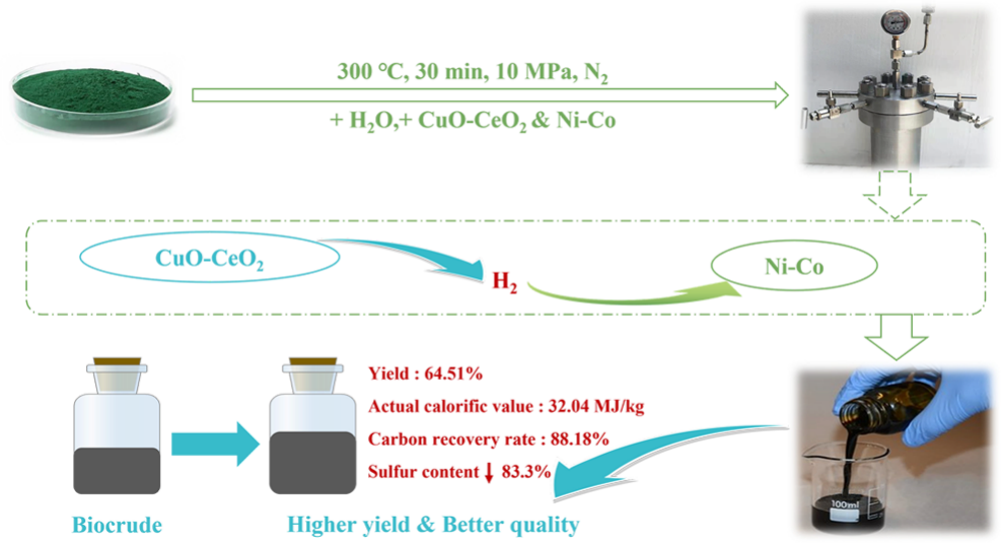

Hydrothermal liquefaction (HTL) is one of the most promising technologies for biofuel production. The preparation and application of catalysts for HTL have been the research focus in recent years. In this study, a new synergistic catalytic process strategy is proposed. CuO–CeO_2_/γ-Al_2_O_3_ was used as an in situ hydrogen donor catalyst and Ni–Co/SAPO-34 was synthesized for hydroprocessing to improve bio-oil production process. The results of XRD and XPS demonstrated that the metal components were well supported on the catalyst. When the two catalysts were mixed, the yield of bio-oil increased from 51.00% to 64.51%, the carbon recovery rate raised from 69.53% to 88.18%, the energy recovery rate grew from 63.42% to 80.22%, and the S content is relatively reduced by 83.3%. Also, TG analysis showed that the content of light components in bio-oil increased. Moreover, the hydrocarbons and alcohols were observed to a higher proportion from the GC-MS analysis. This new method still has high catalytic activity after repeated use for five times. This study provides a new idea for preparing higher yield and superior quality bio-oil.

## 1 Introduction

Countries around the world are facing the following problems in the field of energy and environment:^1,2^ (1) consumption of nonrenewable energy, (2) reduction of conventional energy supply, (3) potential energy security crisis in geopolitical conflicts, (4) climate change caused by greenhouse gas emissions. The existence of these problems has prompted the global research on renewable energy in the past few decades. Among them, biomass plays a crucial role in the production of biofuels and derived chemicals to complement existing fossil fuels and their derivatives.^3^ Microalgae has the following advantages as third-generation biomass raw materials: fast growth rate, high production efficiency per unit area, relatively less affected by seasonal changes, no competition with food crops for arable land resources.^4,5^ Also, the cultivation of microalgae can reduce greenhouse gas emissions and diminish nitrogen pollutants in water.^6^

Hydrothermal liquefaction is a commonly used thermochemical conversion process which can convert biomass into biobased fuels or value-added chemicals without pretreatment.^7−9^ Spirulina with high protein content is a commonly used HTL material, which is widely used in catalytic HTL,^10^ coliquefaction upgrading in batch reactors,^11^ and pilot-scale continuous reactor.^12^ However, the yield and quality of bio-oil cannot meet commercial operation under normal conditions.^13^ Catalytic hydrodeoxygenation (HDO) has been proven to be an effective method for upgrading petroleum, pyrolysis bio-oil, and HTL biocrude. The use of catalysts can improve the quality or yield of bio-oil by reducing the activation energy of the reaction. Pt,^14^ Pd,^15,16^ and Ru^17^ have been widely used to upgrade bio-oil due to their antisintering ability and more acid sites. However, the application of these precious noble metals is hindered by their high costs. Transition metals (e.g., Ni, Co, Mo, Cu, and Ce) are very active catalysts for promoting the re-formation of some components in the bio-oil to hydrocarbons.^18−20^ As an inexpensive catalyst, the supported nickel catalyst has good performance in increasing the yield of bio-oil and significantly reducing the content of O, N, and S heteroatoms.^21,22^ The modified nickel-based catalyst has a better effect. With the synergistic metal effects, several properties of Ni-based catalysts can be acquired such as higher Ni dispersion and higher direct deoxygenation selectivity.^23^ Muangsuwan et al.^24^ modified Ni/Al_2_O_3_ and Co/Al_2_O_3_ with Mo and found that the addition of Mo increased the yield of bio-oil in 350 °C. Raikwar et al.^25^ found that Ni–Co/γ-Al_2_O_3_ demonstrated distinct reactivity and selectivity in guaiacol HDO compared to monometallic Ni and Co catalysts. Cu and Ce catalysts are also frequently used in bio-oil upgrading and have synergistic effects under the high-temperature and -pressure conditions. Ma et al.^26^ demonstrated that CeO_2_ modified by Cu could be effective in improving the bio-oil yield and selectivity in the HTL process of rice straw.

The current conventional hydrodeoxygenation of bio-oil requires additional H_2_ atmosphere.^27^ This limits the practical application of bio-oil upgrading due to higher parameter requirements for production equipment and higher operating costs. In situ hydrogen donation from catalysts in subcritical or supercritical water (sub-CW or SCW) reaction environments is another attractive method for catalytic deoxygenation.^28,29^ In our previous study,^30^ it was confirmed that CuO–CeO_2_/γ-Al_2_O_3_ has the role of donating hydrogen in the reaction system through the experimental results and the calculation of density functional theory (DFT). Under the action of CuO–CeO_2_/γ-Al_2_O_3_, the activation energy of carboxylic acid decarboxylation reduced from 24.8 kcal mol^–1^ to 15.0 kcal mol^–1^, which meant in situ H_2_ supply could occur more easily through water-shift reaction. Although this catalyst works well in the catalytic reaction of stearic acid, its specific performance in bio-oil needs to be further verified.

Meanwhile, although the catalytic hydrodeoxygenation process has been extensively studied in the past few years, only less studies pointed out the synergistic effect of various heterogeneous or homogeneous catalysts for HTL. According to a study by Wang et al.,^31^ the optimal HHV and yield are obtained when NaOH and Raney nickel are used synergistically to catalyze the HTL of lignin. Chen et al.^32^ found that the synergistic use of Na_2_CO_3_ and Fe increased the yield of bio-oil by about 32%. However, few studies have focused on the effect between metal-supported heterogeneous catalysts.

This work aims to explore the synergistic effect of metal-supported catalysts. Another purpose is to verify the feasibility of in situ hydrogen supply for hydrodeoxygenation in the HTL process of biomass. In this work, CuO–CeO_2_/γ-Al_2_O_3_ and Ni–Co/SAPO-34 and their mixture were prepared for HTL of Spirulina. The catalysts were characterized by XRD and XPS. The effect of catalysts on bio-oil yield, elemental composition, and calorific value was investigated. Molecular composition characterization of bio-oil was obtained by gas chromatography–mass spectrometry (GC-MS). The boiling point range of bio-oil was analyzed by thermogravimetric analysis (TGA). In order to evaluate the industrial application potential of this new method, we also examined its reusability.

## 2 Materials and Methods

### 2.1 Materials

Spirulina powder produced by Shandong Binzhou Tianjian Biotechnology Co. is used as raw material, and its characteristic is shown in Table 1. Cu(NO_3_)_2_·6H_2_O and Ce(NO_3_)_3_·6H_2_O were purchased from Shanghai Macklin Biochemical Co., Ltd. NiCl_2_·6H_2_O and γ-Al_2_O_3_ were purchased from Sinopharm Chemical Reagent Co., Co(NO_3_)_2_·6H_2_O was purchased from Shanghai Aladdin Biochemical Technology Co., Ltd., and SAPO-34 was purchased from Nanjing XFNANO Materials Tech Co., Ltd. All water used in this work is 18 MΩ cm deionized water with Milli-Q. In addition, the purity of the reagents in this experiment was 99.9%.

**Table 1:** Textural Composition and Proximate and Element Analysis of Spirulina Powder

Property	Value
Component Content of Organic Matter (wt %)^a^	
Protein content	65
Lipid content	5.9
Carbohydrate content	10.8
Other organics	4.85
	
Proximate Analysis (wt %)^a^	
Moisture	6.8
Ash	6.65
	
Organic Element Content (wt %)^a^	
C	45.91
H	6.75
N	10.33
S	0.69
O^b^	36.32
	
HHV (MJ kg^–1^)^c^	19.93

a On dry basis.

b Calculated by differences.

c Measured by oxygen bomb calorimeter.

### 2.2 Catalyst Preparation

In this study, CuO–CeO_2_/γ-Al_2_O_3_ (hereafter referred to as Cu–Ce) was prepared by incipient wetness impregnation. The metal salt solution was prepared with copper nitrate and cerium nitrate. Theoretically, the content of CuO and CeO_2_ was 10 wt %. After mixing with the carrier evenly, it was dried at 105 °C for 12 h. The catalyst was ground to below 20 mesh before calcination and then calcined in a muffle furnace at 400 °C for 6 h.

The Ni–Co/SAPO-34 catalyst (hereafter referred to as Ni–Co) was prepared by the liquid-phase reduction method. SAPO-34 was purchased from Sinopharm Group. Before being impregnated with the metal salt solution, it was dried at 105 °C for 2 h, then ground, and sieved to obtain catalyst particles with a size of ≤0.22 mm. NiCl_2_ and Co(NO_3_)_2_ were used o prepare metal salt solution, adding excess NaBH_4_ solution dropwise and stirring, coloading SAPO-34 catalyst with 10 wt % nickel and 10 wt % cobalt, and then drying at 105 °C for 12 h.

### 2.3 HTL Experiment of Spirulina

Bio-oil is prepared by a HTL method. Heating was performed by an external electric furnace using a 500 mL batch reactor (GSH-0.5, Weihai Chemical Machinery Co., Ltd., China). According to the work of Wang et al.,^33^ the operating conditions and experimental procedures for HTL were determined. The amounts 10 wt % γ-Al_2_O_3_, 10 wt % SAPO-34, 10 wt % Cu–Ce, 10 wt % Ni–Co, 5 wt % Cu–Ce, and 5 wt % Ni–Co were added for experimentation, separately, 24.00 g of Spirulina, 120 mL of deionized water were also added, and nitrogen gas was purged for 10 min to remove air in the reactor with/without catalyst. The rotation speed was maintained at 80 rpm Then the reactor was heated from 20 to 300 °C in 40 min and kept at 300 °C for 30 min. The residence time did not include the heating time. After the reaction was completed, the cooling water was turned on and cooled to room temperature. After the pressure was released, the reactor was flushed with CH_2_Cl_2_ (DCM, 300 mL) and then the liquid and solid product were collected. After the product was vacuum filtered through a 0.45 μm filter membrane, the organic phase and the aqueous phase were separated with a separating funnel. The organic phase was rotary-evaporated under negative pressure at 40 °C for 1 h to obtain bio-oil. Each experiment was repeated three times. The gaseous product was collected using a 0.5 L gas sampling bag and weighed before and after collection to calculate the gas weight.

Bio-oil yield (*Y*_Bio-oil_, %), gaseous product yield (*Y*_GP_, %), solid residue yield (*Y*_SR_, %), water-soluble product yield (*Y*_WSP_, %), energy recovery rate (ER, %) and carbon recovery rate (CR, %) were calculated by eqs 1–6:
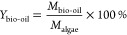
1
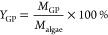
2
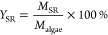
3
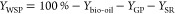
4
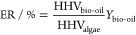
5
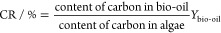
6where *M*_algae_, *M*_bio-oil_, *M*_GP_, *M*_SR_, and *M*_WSP_ are the masses of spirulina raw material, bio-oil, gaseous product, solid residue, and water-soluble product, respectively; HHV (MJ kg^–1^) means the higher heating value of bio-oil or spirulina.

### 2.4 Catalyst Characterization

The X-ray diffraction patterns (XRD) of the catalysts were analyzed by a Rigaku Smartlab SE type ray diffractometer equipped with Cu Kα radiation. Data were collected with the settings of 40 kV and 30 mA at steps of 0.02° s^–1^ in the 2θ range of 10°–80°.

A Thermo Scientific K-Alpha X-ray photoelectron spectrometer (XPS) equipped with an Al Kα excitation source was used to analyze the elemental composition of the solid surface. The spot size was 400 μm, the working voltage was 12 kV, and the filament current was 6 mA.

### 2.5 Characterization of liquid products

Elemental analysis of the bio-oil was performed with a Vario EL cube III elemental analyzer (Elementar, Germany). The content of O was calculated according to the difference method.

GC-MS (5977 A, Agilent) was used to analyze the composition of biocrude. The instrument was equipped with an HP-5 ultrainert chromatographic column (30 m × 250 μm × 0.25 μm). The temperatures of the ion source and the quadrupole were 230 °C and 150 °C, respectively. Helium was used as the carrier gas (flow rate = 1 mL min^–1^). The column temperature initially was set to 60 °C for 1 min, then increased to 70 °C at a rate of 1 °C min^–1^, afterward increased to 300 °C at 10 °C min^–1^, and finally kept at 300 °C for 10 min. It should be noticed that by comparing with the National Institute of Standards and Technology (NIST) database, only light molecules with low boiling points can be measured and only a limited number of compounds could be identified.

The HHV of bio-oil was determined by Sande SDC712 oxygen bomb calorimeter. The boiling point distribution of the bio-oil was analyzed by a thermogravimetric instrument (DTG-60, Shimadzu). Approximately 10 mg of sample in an alumina crucible was heated from 20 to 800 °C at a rate of 10 °C min^–1^. The nitrogen flow rate was 40 mL min^–1^.

## 3 Results and Discussion

### 3.1 Catalyst Characterization

**Figure 1:**
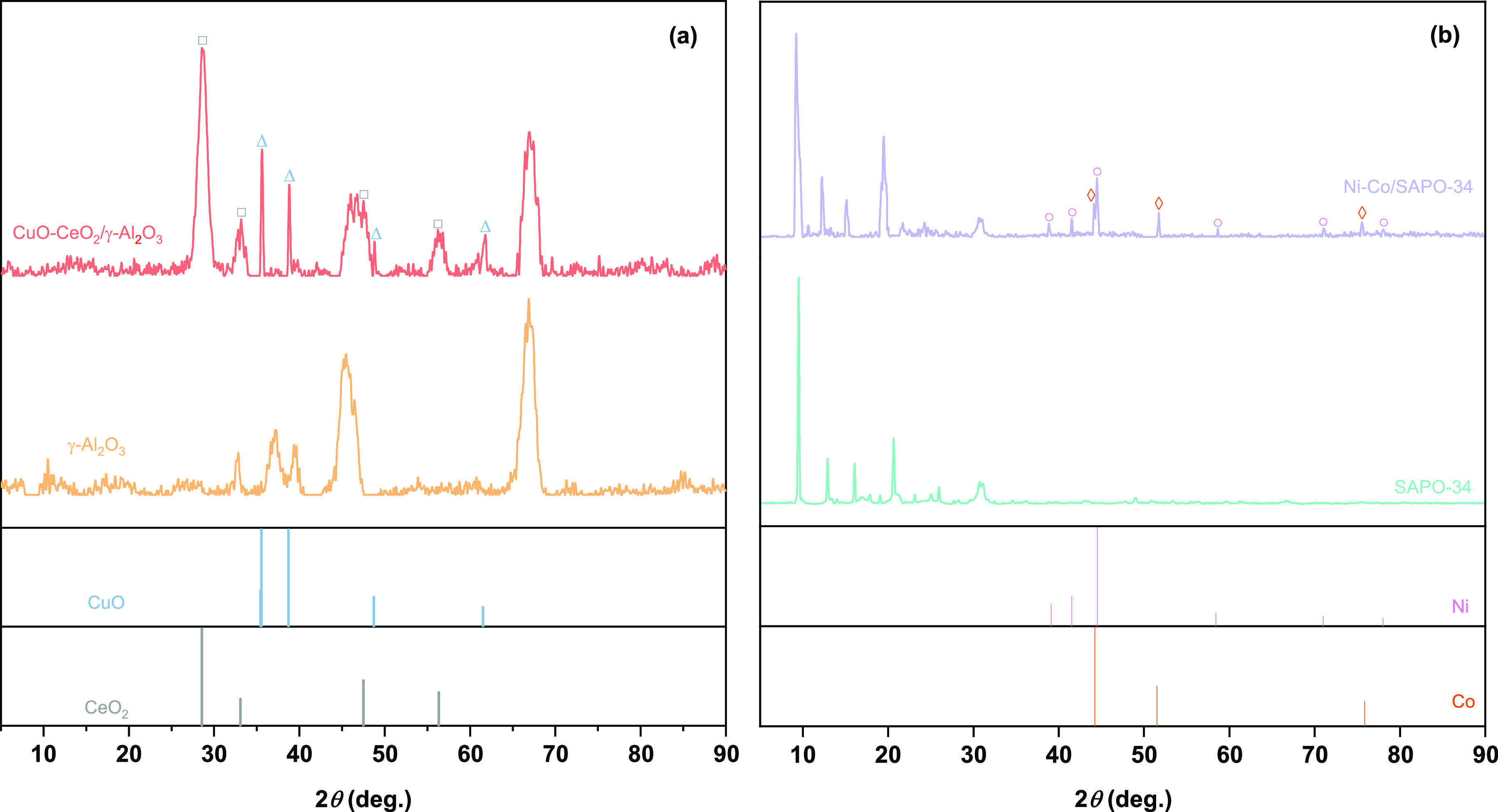
XRD patterns of (a) γ-Al_2_O_3_ and CuO–CeO_2_/γ-Al_2_O_3_ and (b) SAPO-34 and Ni–Co/SAPO-34.

As shown in Figure 1, the crystallographic characteristics of the catalysts were investigated by XRD. The commercial γ-Al_2_O_3_ used as catalyst support has diffraction peaks at 2θ of around 37.6°, 39.3°, 45.7°, and 67°, which correspond to (311), (222), (400), and (440) crystal planes (a face-centered cubic phase) of γ-Al_2_O_3_ with low crystallinity.^34,35^ Peaks were found at 2θ of 35.5°, 38.7°, 48.6°, and 61.5°, which correspond to the (002), (111), (202), and (113) crystal planes of CuO. The peaks of CeO_2_ at 2θ of 28.36°, 32.84°, 47.42°, and 56.34° were also observed. This indicates that Cu and Ce are well supported on the catalyst in the form of CuO and CeO_2_, respectively. Du et al.^30^ also observed diffraction peaks of CuO and CeO_2_ when the loadings of Cu and Ce were 10%. SAPO-34 has sharp characteristic diffraction peaks before loading, which indicates that SAPO-34 has a good CHA topology.^36^ Characteristic peaks for Ni and Co were observed, which indicated that Ni and Co were well dispersed on the surface of the SAPO-34 support.

The elemental composition of the catalyst surface was studied by XPS. The contents of Cu and Ce on the surface of CuO-CeO_2_/γ-Al_2_O_3_ are 0.23% and 0.47%, respectively. It is reasonable that the support γ-Al_2_O_3_ has a better mesoporous structure, and Cu and Ce may exist more in the bulk phase, which can be well understood by the diffraction peaks of CuO and CeO_2_ observed in XRD. Ni and Co were detected on the surface of Ni–Co/SAPO-34, and their contents were 17.5% and 14.0%, respectively. This can be explained by SAPO-34 having more micropores so that Ni and Co are more easily enriched on the catalyst surface. It caused the content of Ni and Co on the catalyst surface to be higher than the theoretical content. It should be noted that XPS is a typical surface analysis method,^37^ which is used for qualitative and semiquantitative analysis. The test area is generally hundreds or even tens of μm on the sample surface at the depth of 1–10 nm, which does not represent the overall properties of the sample. Therefore, the higher contents of Ni and Co may also be due to their uneven distribution on the sample surface.

### 3.2 Effect of Catalysts on Bio-oil Yield

**Figure 2:**
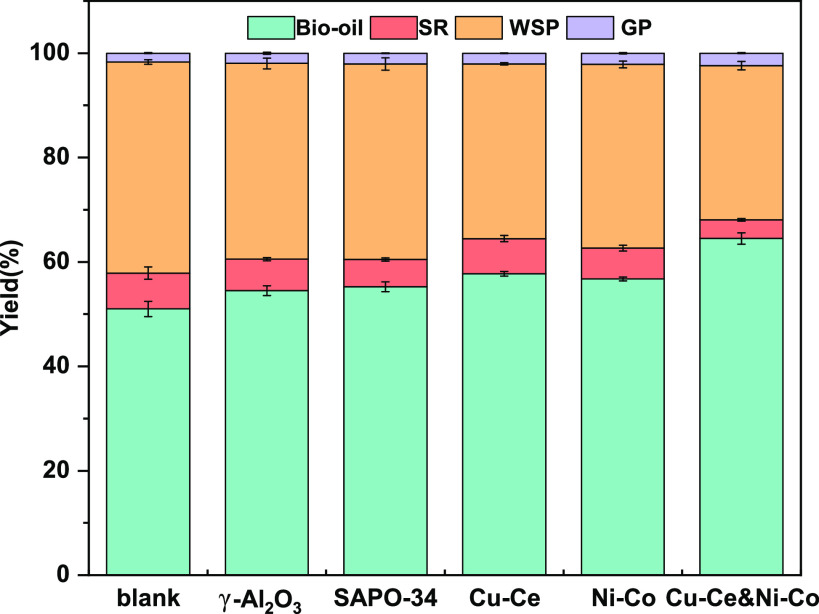
Yield of bio-oil under different catalyst conditions.

Figure 2 shows the effects of different catalyst usage methods on the yield of bio-oil. It is worth noting that the bio-oil yield without catalyst (51.00%) is different from the bio-oil yield obtained by Vardon^11^ and Anastasakis et al.^12^ previously, which is 32.6% and 14.6%–53.6%, respectively. This is due to different biochemical and elemental compositions of spirulina raw materials, different reaction devices (such as agitator and volume), and different methods of bio-oil extraction and separation. Bio-oil in this study actually refers to all DCM-soluble components after HTL. This is similar to the bio-oil yield obtained from some other HTL studies using spirulina as raw material.^33,38^ A lower solid concentration (16.7%) was used in this study. Yang et al.^39^ found that lower solid-to-liquid ratio within a certain range will improve the biocrude productivity. The biochemical components of spirulina became more soluble, resulting in stronger relative interactions between fragments at low solid concentration.

After adding different catalysts, the yield of bio-oil has all been improved to different extents. The influence of the catalyst without metal loading on the bio-oil yield is in the range of 3.49%–4.25%. After loading the metal, the bio-oil yield increased about 5.72%–6.75%, which is similar to the research of Wang et al.^33^ When Cu–Ce and Ni–Co were used together, the yield of bio-oil increased by 13.51%. It means that Cu–Ce and Ni–Co have a distinct synergistic effect in the bio-oil production process. The gaseous product yield remained at about 2 wt % and had little change before and after the catalyst was used. According to the carbon balance of the whole reaction, this new catalytic method (Cu–Ce and Ni–Co) promoted the conversion of water-soluble products and solid products to bio-oil. According to the results of GC-MS, this is because the use of Cu–Ce and Ni–Co promoted the repolymerization of water-soluble products, which led to the increase in the content of higher fatty alcohols, hydrocarbons, and ketones in bio-oil. At the same time, Cu–Ce and Ni–Co further promoted the depolymerization of the biochemical components of spirulina, resulting in less solid residue generation.

### 3.3 Bio-oil Characterization

#### 3.3.1 Elemental Analysis

Table 2 shows the effects of different methods on the elemental composition, HHV and ER of bio-oil. The elemental analysis was carried out twice, and the average value is shown in the table. Compared with the raw material of spirulina, the oxygen content of the bio-oil obtained by hydrothermal liquefaction was significantly decreased and the HHV was significantly increased, which indicated that hydrothermal liquefaction was an effective way to produce liquid fuel. After using Ni–Co catalyst, the S content in bio-oil decreased significantly. Compared with biocrude, the relative reduction ratio of S content reaches 83.3%. The lower sulfur content makes bio-oil more competitive as biofuel.^40^ This can be attributed to the addition of Co to the Ni–Co catalyst that reduces the electron donating properties from d-orbital, altering the activity of the catalyst and prompting the migration of S into the water or gas phase. It may also be because unvulcanized Co reacts with S in the bio-oil, resulting in a decrease in S content and catalyst inactivation.

**Table 2:** Changes in Element Content, Carbon Recovery Rate (CR, wt%), HHV (MJ kg^–1^), and Energy Recovery Rate (ER,%) of Bio-oil Obtained under Different Conditions

	N (%)	C (%)	H (%)	S (%)	O (%)	O/C	H/C	HHV	CR (%)	ER (%)
Spirulina	10.33	45.91	6.75	0.69	36.32	0.79	0.15	19.93		
Biocrude	8.83	62.59	8.10	0.24	20.24	0.32	0.13	31.03	69.53	63.42
γ-Al_2_O_3_	8.23	64.62	6.89	0.31	19.86	0.31	0.11	31.14	76.69	67.76
SAPO-34	7.78	64.22	6.91	0.38	20.62	0.32	0.11	31.48	77.29	68.71
Ni–Co	7.97	61.42	6.76	0.04	23.72	0.39	0.11	30.76	75.88	70.54
Cu–Ce	8.03	65.58	7.20	0.23	18.96	0.29	0.11	32.19	82.50	71.82
Cu–Ce and Ni–Co	8.24	62.76	6.85	0.04	22.11	0.35	0.11	32.04	88.18	80.22

When Ni–Co is used alone, the O content increases slightly. When Cu–Ce is used alone, the O content decreases slightly. The effect of oxygen content is moderated and the actual calorific value of bio-oil will be slightly improved when the two catalysts are used synergistically. Due to the increase of bio-oil yield with the same amount of raw material, the energy recovery rate of the whole process changed from 63.42% to 80.22%, which is a very obvious improvement compared to the energy recovery rate without catalyst. The carbon recovery rate increased from 69.53% to 88.18%, which indicated that most of the carbon elements in spirulina were transferred to the bio-oil.

#### 3.3.2 GC-MS Analysis

**Figure 3:**
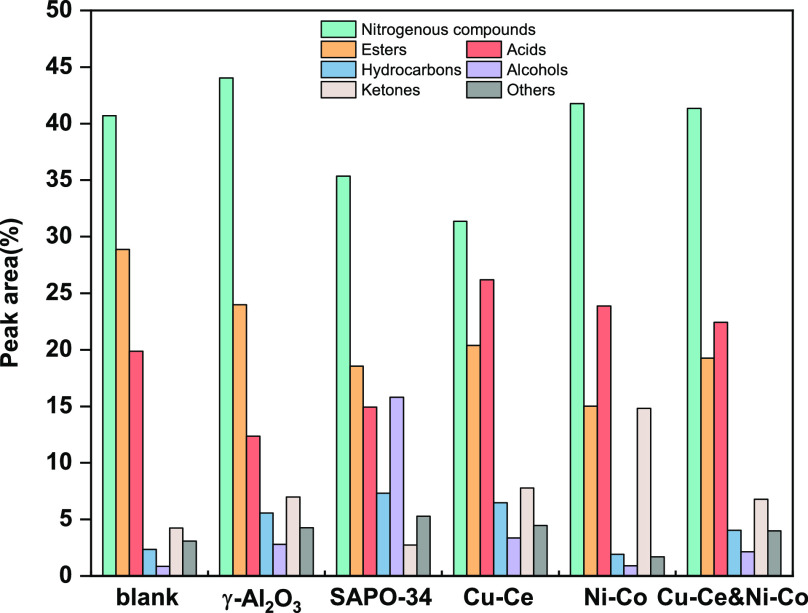
GC-MS analysis of bio-oil under different catalyst conditions.

The component content of bio-oil is shown in Figure 3. It can be seen that bio-oil is mainly composed of nitrogenous compounds, esters, acids, hydrocarbons, alcohols, ketones, etc., which is consistent with previous studies.^41,42^ Esters are produced from the reaction between the fatty acids with the alcohols. The use of catalysts can reduce the content of esters in bio-oil. This may be because the catalyst promotes the conversion of acids or alcohols to ketones and indirectly inhibits the esterification reaction. SAPO-34 can effectively promote the formation of alcohols and hydrocarbons and reduce the content of nitrogenous compounds. Ni–Co can promote the conversion of esters to acids and ketones.

When the two catalysts are used synergistically, the conversion of esters to other components is promoted. The effects of the dual catalysts on nitrogen-containing compounds, hydrocarbons, alcohols, and esters are in the middle of the effects that Cu–Ce or Ni–Co can produce alone. It should be noted that the synergistic use of the dual catalysts promoted further conversion of acids and ketones. Compared with the blank experiment without catalyst, the content of hydrocarbons and alcohols in the collaborative catalytic system was slightly increased, and the content of esters decreased significantly. The hydrocarbons mainly come from the decarboxylation and cracking of fatty acids. The nitrogenous compound content increased slightly. It is because the repolymerization of amino acids formed by protein hydrolysis in the aqueous phase was promoted when both catalysts are used together, which is also one of the reasons for the increase in bio-oil yield.

#### 3.3.3 Thermogravimetric Analysis

**Figure 4:**
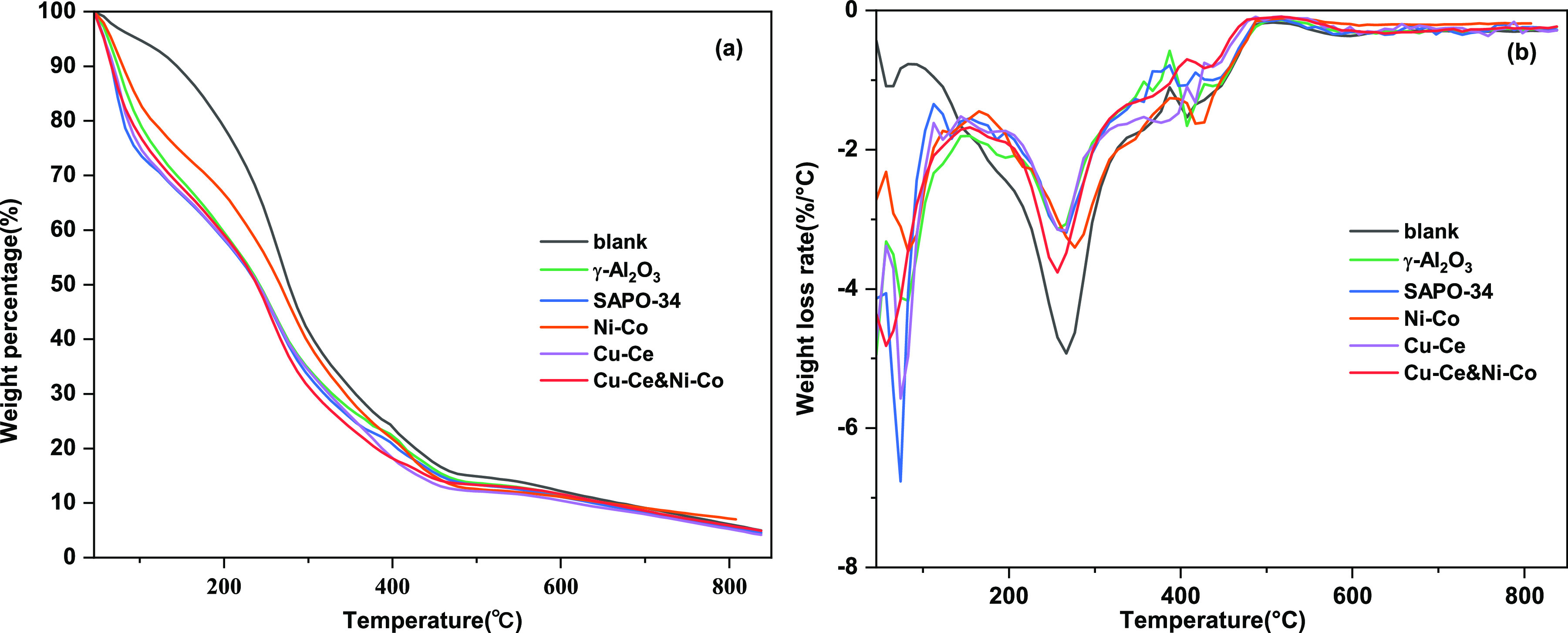
TG (a) and DTG (b) curves of the bio-oil over different catalysts.

Thermogravimetric analysis of the bio-oil was performed to determine its thermal stability, and the results were shown in Figure 4. It can be clearly seen that the bio-oil is more volatile after using the catalyst. This indicates that the catalyzed bio-oil has more low-boiling components. The distilling ranges of gasoline and diesel were 30–220 °C and 180–410 °C, respectively. It can be seen that when the two catalysts are used synergistically, the volatilization ratio of bio-oil is 82.76% at 400 °C, which indicates that the bio-oil under this condition has better potential to be used as biofuel. It can be seen from the DTG curve of bio-oil that the catalyzed bio-oil has a faster weight loss rate in the ranges of 70–90 °C and 240–270 °C. The weight loss rate of the biocrude without catalyst was faster in the range of 240 °C–270, indicating that the use of catalyst promoted the conversion of the 240 °C–270 °C component to the 70 °C–90 °C component. This can be explained by nickel favoring successive hydrogenolysis of C–C bond,^23^ leading to ring opening of furans or formation of shorter chained hydrocarbons at high temperature.

**Figure 5:**
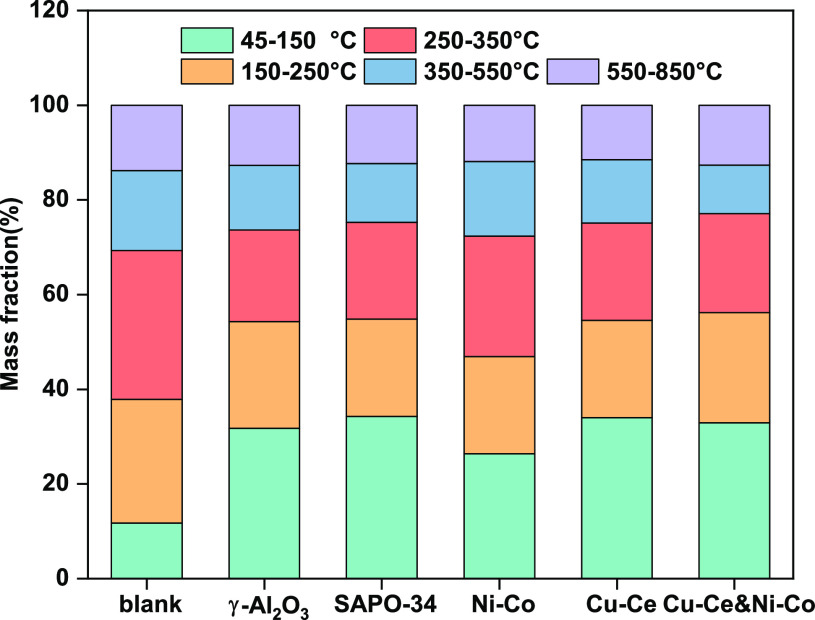
Boiling point range distribution of the bio-oil under different catalyst conditions.

TG analysis under nitrogen atmosphere can be used to estimate the boiling point range of different components of bio-oil.^43^Figure 5 shows the boiling range distribution of bio-oil prepared under different catalyst conditions. Compared with the biocrude without catalyst, the catalyzed bio-oil has more components below 150 °C. All kinds of bio-oil have similar component contents above 350 °C. It can be deduced that the use of the catalyst promotes the conversion of the 250 °C–350 °C components to light components. After 500 °C, the remaining components of bio-oil are asphaltene, which is difficult to volatilize, and even coking may occur. When the two catalysts are used together, the content of components below 350 °C in the bio-oil is the highest, reaching 77.10%, and the content of heavy components is the lowest. It was observed in the experiment that the fluidity of the prepared bio-oil was the best at this turn, which was consistent with the conclusion drawn from the TG analysis.

### 3.4 Reusability of Catalysts

**Figure 6:**
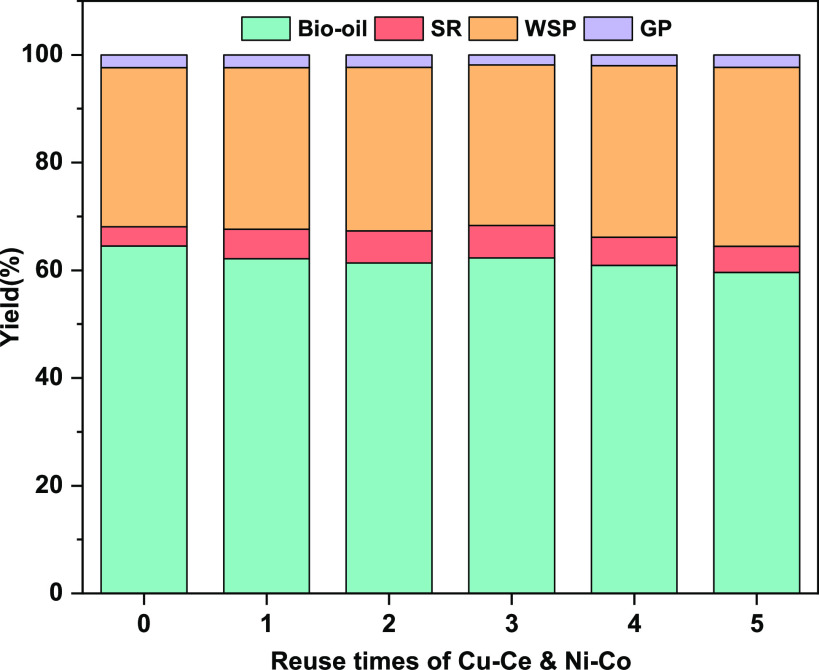
Products yield under different reuse times of Cu–Ce and Ni–Co.

In addition to catalytic activity, catalyst recoverability and reusability are also important challenges for industrial application.^44^ Cu–Ce and Ni–Co can be easily collected by phase separation technology after the reaction because they are heterogeneous catalysts. The traditional calcination method will change the activity of the Ni–Co catalyst. Considering the low solid residue yield in the HTL reaction of spirulina, all dried solid products, including catalysts and solid residue, are reused in the reusability tests. The effect of catalysts reuse is shown in Figure 6. It can be seen that the catalysts still have good catalytic activity after repeated use for five times. Compared with the blank group experiment, the bio-oil yield still increased by 8.63% in the fifth cycle. However, the catalytic activity of the catalysts decreased observably with the increase of recycling times. This is probably due to the influence of solid residue accumulated in the recycling experiments. If this new catalytic method is applied in the fixed bed reactor, the solid residue can be separated in time, and the catalytic activity may be maintained for more reuse times theoretically.

## 4 Conclusion

Hydrothermal liquefaction is a promising technology that can produce biofuels and value-added chemicals for partially replacing petroleum. Improving the yield of bio-oil and reducing the content of heteroatoms in bio-oil are prerequisites for commercial operation. In this study, a synergistic catalytic strategy was designed based on the different functions of the two catalysts. We found that the yield of bio-oil increased from 51.00% to 64.51%, the actual calorific value was 32.04 MJ kg^–1^, and the carbon recovery rate reached 88.18% and the content of S decreased by 83.3% when Cu–Ce was used in conjunction with Ni–Co. The water-soluble product and solid residue further transform into bio-oil when the two catalysts are used together. This may be attributed to the promotion of Cu–Ce and Ni–Co on the depolymerization of spirulina and the repolymerization of water-soluble products. Meanwhile, the content of light components in bio-oil increased, and the proportion of hydrocarbons and alcohols increased slightly. These advantages make the bio-oil prepared from spirulina have better biofuel potential. However, nitrogen and oxygen in bio-oil still need to be further removed. Reuse testing shows that Cu–Ce and Ni–Co still have high catalytic activity after repeated use for five times. This study provides a new idea for preparing higher yield and superior quality bio-oil.
